# Monocytic Cytokines in Autoimmune Polyglandular Syndrome Type 2 Are Modulated by Vitamin D and HLA-DQ

**DOI:** 10.3389/fimmu.2020.583709

**Published:** 2020-12-07

**Authors:** Anna U. Kraus, Marissa Penna-Martinez, Firouzeh Shoghi, Gesine Meyer, Klaus Badenhoop

**Affiliations:** Division of Endocrinology, Diabetes and Metabolism, Department of Internal Medicine I, University Hospital Frankfurt, Frankfurt am Main, Germany

**Keywords:** Addison’s disease, type 1 diabetes, autoimmune thyroiditis, autoimmune polyglandular syndrome type 2, cytokine gene expression, HLA DQ haplotypes

## Abstract

**Context:**

Autoimmune polyglandular syndrome (APS-2: autoimmune Addison’s disease or type 1 diabetes) is conferred by predisposing HLA molecules, vitamin D deficiency, and heritable susceptibility. Organ destruction is accompanied by cytokine alterations. We addressed the monocytic cytokines of two distinct APS-2 cohorts, effects of vitamin D and HLA DQ risk.

**Methods:**

APS-2 patients (n = 30) and healthy controls (n = 30) were genotyped for HLA DQA1/DQB1 and their CD14+ monocytes stimulated with IL1β and/or 1,25(OH)_2_D_3_ for 24 h. Immune regulatory molecules (IL-6, IL-10, IL-23A, IL-15, CCL-2, PD-L1), vitamin D pathway gene transcripts (CYP24A1, CYP27B1, VDR), and CD14 were analyzed by enzyme-linked immunosorbent assay and RTqPCR.

**Results:**

Pro-inflammatory CCL-2 was higher in APS-2 patients than in controls (p = 0.001), whereas IL-6 showed a trend – (p = 0.1). In vitro treatment with 1,25(OH)_2_D_3_ reduced proinflammatory cytokines (IL-6, CCL-2, IL-23A, IL-15) whereas anti-inflammatory cytokines (IL-10 and PD-L1) rose both in APS-type 1 diabetes and APS-Addison´s disease. Patients with adrenal autoimmunity showed a stronger response to vitamin D. Expression of IL-23A and vitamin D pathway genes VDR and CYP27B1 varied by HLA genotype and was lower in healthy individuals with high-risk HLA (p = 0.0025; p = 0.04), while healthy controls with low-risk HLA showed a stronger IL-10 and CD14 expression (p = 0.01; p = 0.03).

**Conclusion:**

1,25(OH)_2_D_3_ regulates the monocytic response in APS-2 disorders type 1 diabetes or Addison´s disease. The monocytic cytokine profile of individuals carrying HLA high-risk alleles is proinflammatory, enhances polyglandular autoimmunity and can be targeted by vitamin D.

## Introduction

Vitamin D deficiency is found in autoimmune disorders including type 1 diabetes (1–3). The nuclear vitamin D receptor (VDR) and the vitamin D activating enzyme 1-α hydroxylase (encoded by *CYP27B1*) are expressed in monocytes, macrophages, T-, and B- lymphocytes (4, 5). Vitamin D has immune regulatory properties by suppressing proinflammatory cytokines interleukin (IL)-6, IL-2, IL-23, TNFα, IL-12 (6) while stimulating anti-inflammatory cytokines IL-10 and programmed death ligand 1 (PD-L1) (4). This shifts the T helper (Th)1 profile towards a predominant Th2 phenotype and creates a tolerogenic profile (7, 8).

**Figure 1:**
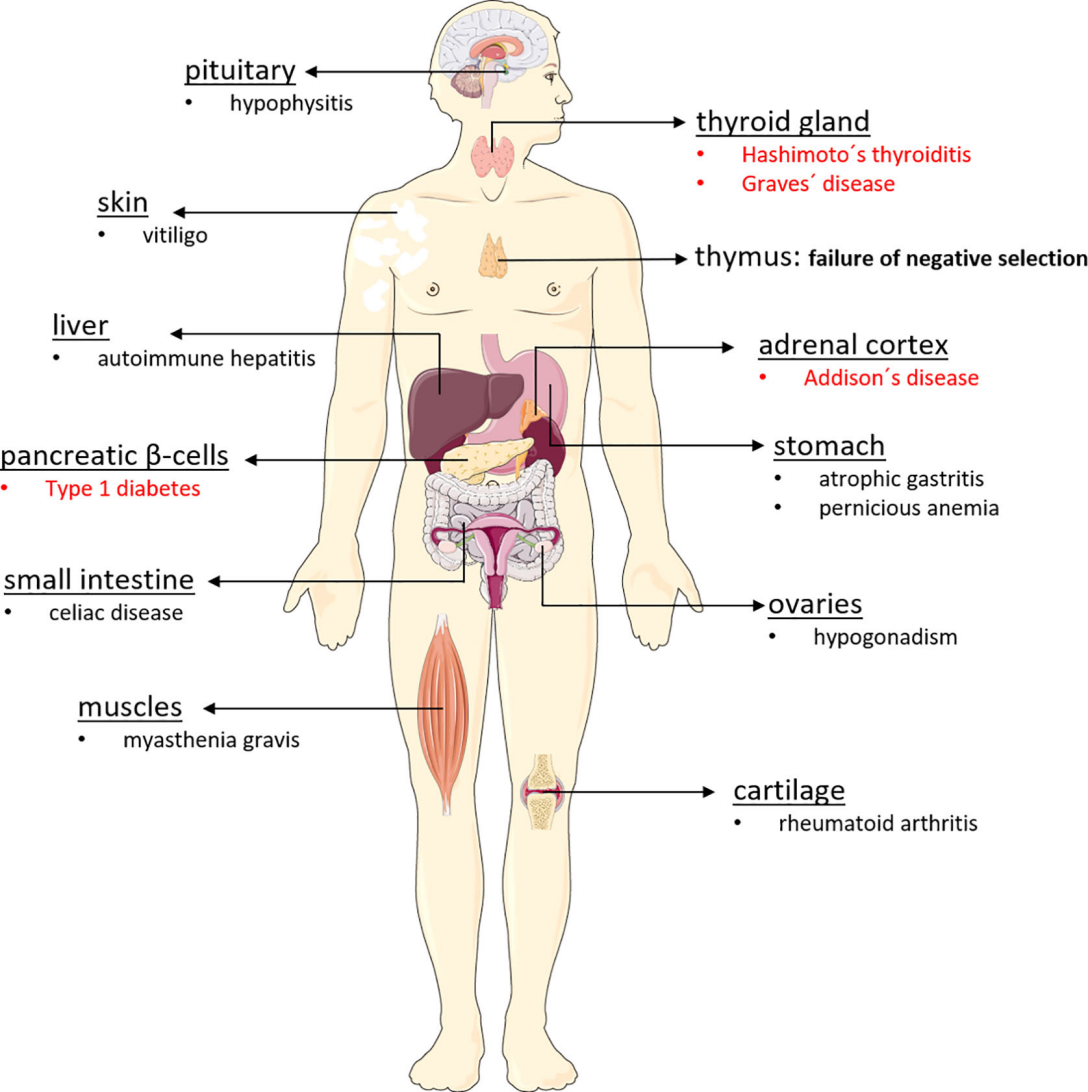
Organs affected in autoimmune polyglandular syndrome type 2. The autoimmune polyglandular syndrome type 2 can affect a wide variety of organs in the body, triggering various autoimmune diseases. For reasons that are still unclear, APS-2 destroys particularly endocrine organs, leading to the development of type 1 diabetes, Addison’s disease, Hashimoto’s thyroiditis, and Graves’ disease. However, non-endocrine organs such as the skin, the gastrointestinal tract, the brain, the liver, and also muscles and cartilage may be affected by the body’s own destruction, which is often driven by antibodies and autoreactive lymphocytes.

Endocrine diseases of the autoimmune polyglandular syndrome type 2 (APS-2) manifest when immune tolerance to endocrine self-antigens is lost, resulting in irreversible selective organ dysfunction. Familial clustering is linked to the major histocompatibility complex class II (MHC II) genes in carriers of HLA-DQA1 and DQB1 alleles DQA1*05-DQB1*02 (DQ2) and DQA1*03-DQB1*0302 (DQ8) (9–11). Dysfunction and mostly destruction of endocrine glands such as the ß-cells of pancreatic islets, adrenal cortex, and thyroid gland results in type 1 diabetes (T1D), Addison’s disease (AD) and autoimmune thyroiditis (AIT) either by hypothyroidism (Hashimoto’s thyroiditis, HT or hyperthyroidism (Graves’ disease, GD) (12, 13). Therefore, APS-2 is characterized by Addison’s disease plus autoimmune thyroiditis (Schmidt syndrome) and/or type 1 diabetes (Carpenter syndrome). Some APS-2 components manifest in childhood, but the syndrome is usually diagnosed in adults when the second APS-2-defining disorder is detected. Besides hormone secreting glands, other tissues are also affected (**Figure 1**).

We investigated cytokine profiles of monocytes as precursors of macrophages and dendritic cells (DC) (14, 15). Besides inhibiting inflammatory cytokines IL-2 and TNF-α (15), vitamin D suppresses monocytes’ differentiation into mature DC, thereby reducing the number of antigen-presenting cells and T cell activating cells, thus inducing both innate and adaptive immune tolerance (14, 16, 17). These immunomodulatory effects have been successfully applied to experimental T1D models by protecting pancreatic β-cells from cytokine-induced inflammation and destruction (18–20).

While 1,25(OH)_2_D_3_ is catalyzed by mitochondrial 1α-hydroxylase (CYP27B1), degradation, and inactivation of both 25(OH)D_3_ and 1,25(OH)_2_D_3_ is catalyzed by 24-hydroxylase (CYP24A1). By hydroxylation of the side chains CYP24A1 promotes the degradation into the water-soluble calcitroic acid and therefore preventing vitamin D toxicity. In the last decades, several studies illustrated the impact of HLA haplotypes and also of vitamin D on immunity independently from each other. Vitamin D was found to modulate excessive MHC II- and antigen expression (21) and healthy subjects homozygous for the HLA high-risk haplotypes showed increased surface expression of HLA-DR and -DQ, and peripheral blood mononuclear cells with increased IL1β and IFN-γ (22). However, genetic susceptibility to APS-2 is conferred by HLA risk alleles, its interaction with the vitamin D system ill-defined. Only one Finnish study finds low vitamin D associated with the HLA B44 supertype (23), but not with HLA-DRB1 or HLA-DQB1 alleles. We therefore addressed this interaction by investigating *in-vitro* effects of active vitamin D 1,25(OH)_2_D_3_ (also referred to as calcitriol) on vitamin D pathway gene transcripts (CYP24A1, CYP27B1, VDR) and monocytic cytokines in patients with APS-2 and healthy controls in correlation with presence or absence of HLA risk heterodimers DQ2 and DQ8.

## Subjects and Methods

### Subjects

A total of 15 T1D patients (9 female/6 male) and 15 AD patients (12 female/3 male) were recruited from the endocrine outpatient clinic of the University Hospital Frankfurt am Main, Germany. All patients were additionally affected by autoimmune thyroiditis (AIT) forming two variants of the APS-2. In AD/AIT patients mean age (± SD) was 57.5 years (± 13.7) and mean age of T1D/AIT patients was 51.5 years (± 13.6). Healthy controls (HC, n = 30, 15 female/15 male) were volunteer blood donors without personal or a family history of autoimmune diseases and were randomly recruited from staff personnel or medical students from the University Hospital. Mean age (± SD) of HC was 41.1 years (± 12.6).

### Monocyte Isolation and Cultivation

The monocyte isolation kit (Ord. no 130-091-153; Miltenyi Biotech, Bergisch Gladbach, Germany) was used for negatively selecting the CD14^+^ monocytes from all subjects. Monocytes were cultured for 24 h in RPMI 1640 medium + L-glutamine (Gibco^®^ Thermo Fisher Scientific) supplemented with 10% heat-inactivated fetal bovine serum (Gibco^®^ Thermo Fisher Scientific) and 100 IU/ml penicillin/streptomycin (Sigma-Aldrich, Taufkirchen, Germany). Monocytes were treated for 24 h, either with 500 IU/ml human IL1β (Ord. no 130-093-897; Miltenyi Biotech) as an inflammatory stimulant, and/or 1 × 10^−8^ mol/L 1,25(OH)_2_D_3_ (Enzo, Lörrach, Germany) for demonstrating the vitamin D-regulating effect in stimulated monocytes, or 1 × 10^−8^ mol/L 1,25(OH)_2_D_3_ was added alone without IL1β, in comparison to untreated cells, for demonstrating the vitamin D-regulating effect in resting monocytes. Dose and exposure time of 1,25(OH)_2_D_3_ and IL1β was based upon our previous work (24).

### RNA Extraction, Reverse Transcription, and Quantitative PCR in Real Time (RT-qPCR)

RNA was extracted using the RNeasy Mini Kit (Cat. no. 74104; Qiagen, Hilden, Bayern), according to the manufacturer´s instructions. For reverse-transcription, 30 ng RNA was transcribed in accordance with instructions of Affinity Script QPCR Kit (Cat. no. 600559; Agilent Technologies) and the resulting cDNA was stored at -80°C. Specific Taqman assay primers (Thermo Fisher Scientific) and qPCR Rox Mix (Cat. no. AB-1138; Thermo Fisher Scientific, Schwerte, Germany) were used to perform the RT-qPCR assays. Primers for genes of the vitamin D pathway (CYP24A1 Hs00167999_m1, CYP27B1 Hs00168017_m1, and VDR Hs01045843_m1), genes encoding for immunological important cytokines and chemokines (IL-23A Hs00900828_g1, CCL-2 Hs00234140_m1, IL-6 Hs00985639_m1, IL-10 Hs00961622_m1, IL-15 Hs01003716_m1, CD274 (PD-L1) Hs01125301_m1) and the gene encoding the monocytic marker CD14 (Hs02621496_s1) were analyzed and compared to gene expression of the house-keeping reference control 18sRNA (Hs99999901_s1). Gene expression levels were quantified by ABI 7300 PCR system (Thermo Fisher Scientific) and the relative transcription levels were analyzed using the comparative cycle threshold (CT), as means of relative quantification, normalized to endogenous reference 18sRNA and expressed as 2^-ΔCT^x10^6^.

The experimental part regarding IL-6 und CCL-2 mRNA expression, as well as CD14 expression in 13 AD/AIT patients, has been published (24) and is therefore not repeated here.

### Enzyme-Linked Immunosorbent Assay

Enzyme-linked immunosorbent assays (ELISAs) were performed using the monocytic supernatants after cell culture. The supernatants were centrifuged for 7 min at 290 g using a benchtop centrifuge to remove cells and cell debris before storage at -80°C. The secreted proteins of IL-6 (Cat. no. 88-7066; RRID : AB_2574993), IL-10 (Cat. no. 88-7106; RRID : AB_2575001), IL-23 (Cat. no. 88-7237; RRID : AB_2575046), IL-15 (Cat. no. 88-7620-88; RRID : AB_2575149), CCL-2 (Cat. no. 88-7399; RRID : AB_2575118)) (all purchased from Thermo Fisher Scientific) and soluble (s) PD-L1 (Cat. no. SEA788Hu, Cloud-Clone Corporation, Texas, USA) were measured according the manufacturer´s instructions with the following exceptions: Supernatants were diluted 1:10 for IL-6 measurements, 1:5 for IL-10 and 1:50 for CCL-2. IL-15, IL-23, and PD-L1 were measured in undiluted supernatant samples. Furthermore, the incubation time of IL-23 and IL-15 was extracted to 20 h at 4°C. The HRP reaction time was conducted for 7 min for IL-6, 14 min for IL-10, 13 min for CCL-2, 20 min for PD-L1, and 15 min both for IL-23 and IL-15. All samples and dilutions of positive and negative control were measured in duplicates.

### HLA DQ Genotyping

Genomic DNA was extracted from blood samples using the salting out method based upon reaction of negatively charged DNA with positively charged sodium. High-resolution sequence specific-primer (SSP) analysis was used for HLA-typing based on template’s 3´ end match or mismatch with PCR-based sequence-specific oligonucleotides. All subjects were genotyped for the MHC class II HLA DQA1/DQB1 alleles DQA1 *0101, *0102, *0103, *0104, *0201, *0301, *0401, *0501, *0601 and DQB1 *0201, *0301, *0302, *0303, *0401, *0402, *0501, *0502, *0503, *0601, *0602, *0603, *0604, using established primers (25). Therefore, all possible α-chain and β-chain variants of DQ were analyzed and subjects were typed according to variants of DQA1 and DQB1 alleles: DQ2 is encoded by the DQB1*0201 and DQA1*0501 alleles, while subjects carrying DQA1*0301 and DQB1*0302 are typed DQ8. Subjects carrying HLA-DQA1*0501 and DQB1*0201 on both loci or DQB1*0201 on both alleles and only one HLA-DQA1*0501 were identified as homozygous for DQ2 (DQ2/DQ2) and subjects with DQA1*0301 and DQB1*0302 on both loci as homozygous DQ8/DQ8. Since DQA1 is in linkage-disequilibrium with DQB1 the extended genotype was determined and categorized in three different risk groups depending on DQ2 and/or DQ8 presence or absence. According to our previous work (10, 24) and congruent findings from others (9, 11) high-risk HLA was defined as HLA homozygous DQ2/DQ2, homozygous DQ8/DQ8 and DQ2/DQ8, heterozygous HLA DQ2/x and HLA DQ8/x as intermediate‐risk HLA and any other haplotype (x/x) as low-risk HLA.

### Statistical Analysis

Statistical significance was determined using Wilcoxon-Mann-Whitney U in group-wise comparisons. The correlation of different treatments within one group was analyzed using the Wilcoxon-matched-paired test. Allele-wise and genotype-wise analysis of polymorphism frequencies were analyzed while stating a null hypothesis in combination with Pearson-Mantel-Haenszels Chi^2^- test, OR and the 95% confidence interval. Analyses were performed using BiAS statistical package version 10.12, here values are considered as significant when p <0.05.

## Results

### Vitamin D Regulates Vitamin D Responsive Genes and CD14 Marker in Primary Monocytes of APS-2 Patients and Healthy Controls

**Figure 2:**
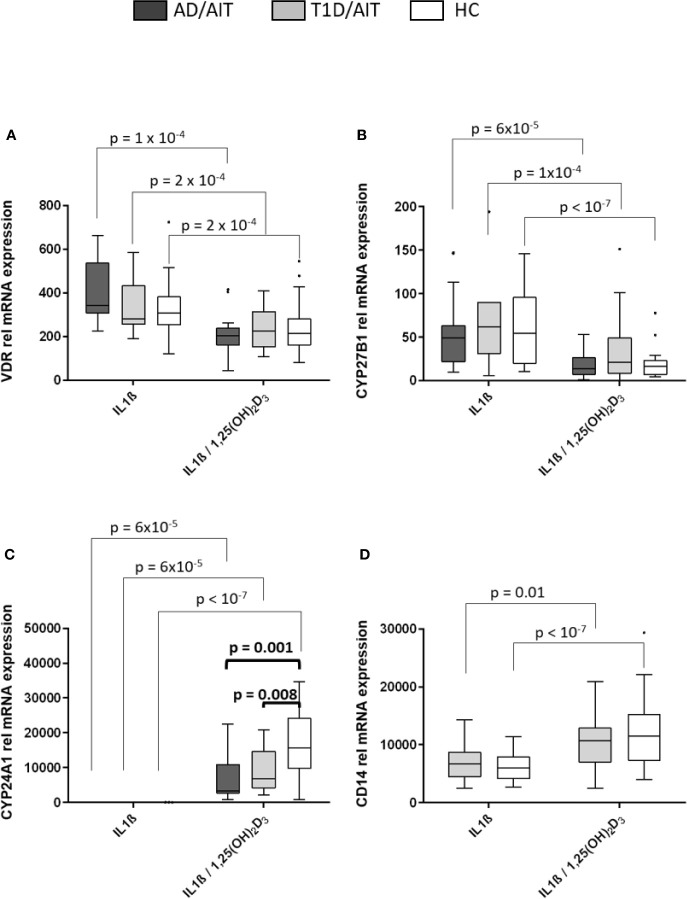
Effect of 1,25(OH)_2_D_3_ on vitamin D pathway genes and CD14 in human monocytes. Effect of 1,25(OH)_2_D_3_ on expression of vitamin D pathway genes **(A–C)** and CD14 **(D)** in IL1β stimulated monocytes of both APS-2 cohorts and HC. Data are presented as median obtained from 15 AD/AIT patients, 15 T1D/AIT patients and 30 healthy controls (HC). T1D, type 1 diabetes; AIT, autoimmune thyroiditis; AD, Addison’s disease; HC, healthy controls. Values ​​are considered to be statistically significant when p <0.05.

Monocytes of patients and of controls showed a marked response to *in-vitro* exposure to 1,25(OH)_2_D_3_. The expression of the VDR and CYP27B1 was induced by IL1β and inhibited after administration of 1,25(OH)_2_D_3_ in both APS-2 and HC (**Figures 2A, B**) [VDR: AD/AIT_IL1β vs IL1β/1,25(OH)2D3_ p = 10^-4^; T1D/AIT_IL1β vs IL1β/1,25(OH)2D3_ p = 2x10^-4^; HC_IL1β vs IL1β/1,25(OH)2D3_ p = 2x10^-4^; CYP27B1: AD/AIT_IL1β vs IL1β/1,25(OH)2D3_ p = 6x10^-5^; T1D/AIT_IL1β vs IL1β/1,25(OH)2D3_ p = 10^-4^; HC_IL1β vs IL1β/1,25(OH)2D3_ p < 10^-7^]. CYP24A1 expression increased in patients and HC following 1,25(OH)_2_D_3_ treatment of IL1β-stimulated monocytes [AD/AIT_IL1β vs IL1β/1,25(OH)2D3_ p = 6x10^-5^; T1D/AIT_IL1β vs IL1β/1,25(OH)2D3_ p = 6x10^-5^; HC_IL1β vs IL1β/1,25(OH)2D3_ p < 10^-7^]. Group wise comparisons reveal differences between APS-2 and HC regarding CYP24A1 mRNA expression. The strong induction of CYP24A1 by 1,25(OH)_2_D_3_ is observed in both APS-2 cohorts, however less than in HC [IL1β/1,25(OH)_2_D_3 AD/AIT vs HC_ p = 0.001, IL1β/1,25(OH)_2_D_3 T1D/AIT vs HC_ p = 0.008] (**Figure 2C**). The expression of VDR or CYP27B1 did not differ between APS-2 and HC.

The monocyte marker CD14 was strongly induced by 1,25(OH)_2_D_3_ in IL1β-activated monocytes in HC and T1D/AIT [T1D/AIT_IL1β vs IL1β/1,25(OH)2D3_ p = 0.01, HC_IL1β vs IL1β/1,25(OH)2D3_ p < 10^-7^] (**Figure 2D**). Also resting monocytes showed an increased CD14 expression upon 1,25(OH)_2_D_3_ (see **Supplementary Material Table A1**).

### Vitamin D Effects on Pro-Inflammatory Gene Expression and Protein Secretion

#### Vitamin D Suppresses Pro-Inflammatory IL-6, CCL-2, IL-23A, and IL-15 mRNA

**Figure 3:**
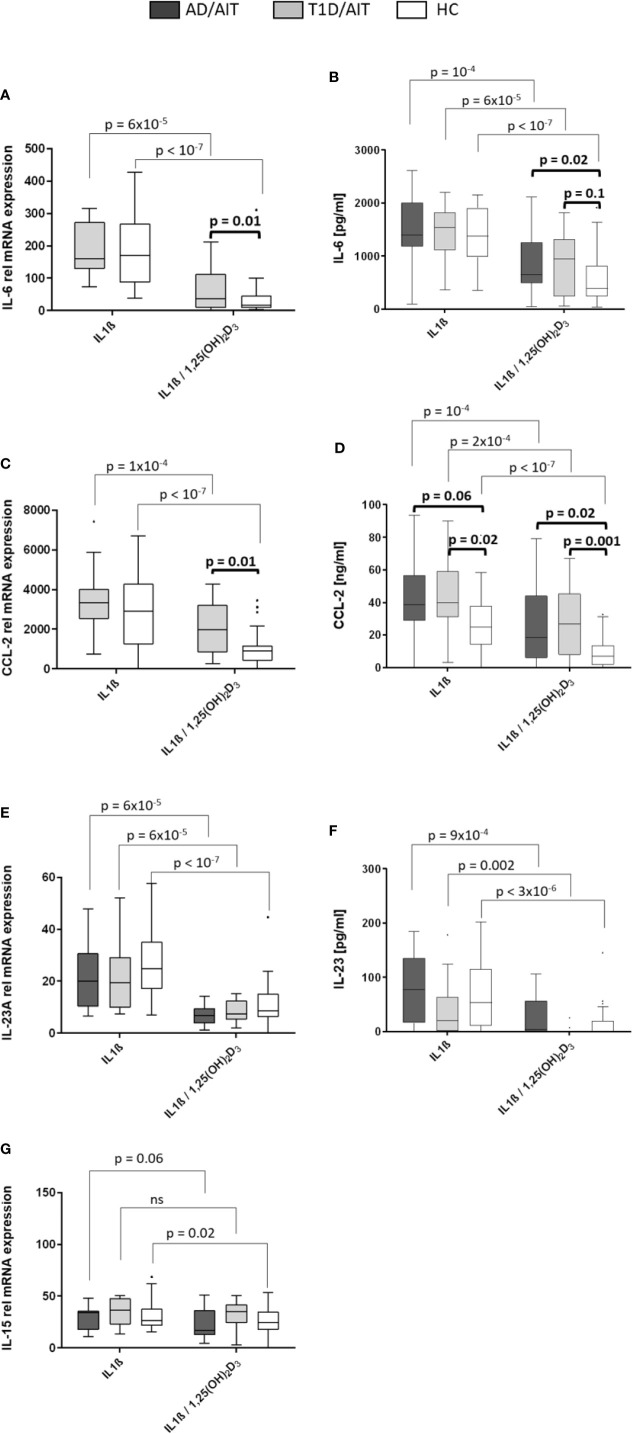
Vitamin D mediated reduction of pro-inflammatory cytokines. Gene expression **(A, C, E, G)** and protein secretion **(B, D, F)** of pro-inflammatory cytokines IL-6, CCL-2, IL-23, and IL-15 after in-vitro 1,25(OH)_2_D_3_ in both APS-2 cohorts and HC. Data are presented as median values obtained from 15 AD/AIT patients, 15 T1D/AIT patients and 30 healthy controls (HC). T1D, type 1 diabetes; AIT, autoimmune thyroiditis; AD, Addison’s disease; HC, healthy controls. Values ​​are considered to be statistically significant when p <0.05.

Inflammatory cytokines were assessed after 24 h of stimulating primary human monocytes at the mRNA level (**Figures 3A, C, E, G**). All pro-inflammatory cytokines were suppressed in HC by the vitamin D metabolite after monocytes had been pre-treated by IL1β. For IL-6-, CCL-2-, and IL23A gene expression this was also found for APS-2 T1D/AIT patients [T1D/AIT: IL-6_IL1β vs IL1β/1,25(OH)2D3_ p = 6x10^-5^, CCL-2_IL1β vs IL1β/1,25(OH)2D3_ p = 10^-4^, IL23A_IL1β vs IL1β/1,25(OH)2D3_ p = 6x10^-5^]. AD/AIT patients showed also decreased IL-23A and IL-15 mRNA levels (by a trend) upon vitamin D addition in IL1β-stimulated monocytes [AD/AIT: IL23A_IL1β vs IL1β/1,25(OH)2D3_ p = 6x10^-5^, IL-15_IL1β vs IL1β/1,25(OH)2D3_ p = 0.06] (**Figures 3E, G**). Group-wise comparison showed increased, but not significantly enhanced IL-6 mRNA of T1D/AIT patients compared to HC [IL1β/1,25(OH)_2_D_3 T1D/AIT vs HC_ p = 0.1]. APS-2 patients have an increased expression of inflammatory cytokines which is pronounced for the CCL-2 expression that strongly differs between T1D/AIT patients and controls: increased levels are found for CCL-2 in IL1β/1,25(OH)_2_D_3_-costimulation [RNA: IL1β/1,25(OH)_2_D_3 T1D/AIT vs HC_ p = 0.01].

#### IL-6 and Chemokine CCL-2 Secretion Suppressed by Vitamin D Exposure

Secretion of pro-inflammatory cytokines upon 24 h treatment of monocytes with IL1β, or IL1β/1,25(OH)_2_D_3_-co-stimulation was measured by ELISA (**Figures 3B, D, F**). The findings of secreted cytokines confirmed the mRNA data: vitamin D suppressed IL-6- and CCL-2 secretion in IL1β-pretreated monocytes of T1D/AIT patients [IL-6_IL1β vs IL1β/1,25(OH)2D3_ p = 6x10^-5^, CCL-2 _IL1β vs IL1β/1,25(OH)2D3_ p = 2x10^-4^, IL-23A_IL1β vs IL1β/1,25(OH)2D3_ p = 0.002], as well as of AD/AIT [IL-6_IL1β vs IL1β/1,25(OH)2D3_ p = 10^-4^, CCL-2_IL1β vs IL1β/1,25(OH)2D3_ p = 10^-4^, IL-23A_IL1β vs IL1β/1,25(OH)2D3_ p = 9x10^-4^], and of HC [IL-6_IL1β vs IL1β/1,25(OH)2D3_ p < 10^-7^, CCL-2_IL1β vs IL1β/1,25(OH)2D3_ p < 10^-7^, IL-23A_IL1β vs IL1β/1,25(OH)2D3_ p = 3x10^-6^]. Group-wise comparisons between APS-2 and HC revealed increased, but not significantly enhanced IL-6 protein in T1D/AIT patients after co-stimulation with IL1β/1,25(OH)_2_D_3_ [IL1β/1,25(OH)_2_D_3 T1D/AIT vs HC_ p = 0.1] and significantly increased IL-6 protein in AD/AIT patients after IL1β/1,25(OH)_2_D_3_-co-stimulation [IL1β/1,25(OH)_2_D_3 T1D/AIT vs HC_ p = 0.02].

CCL-2 secretion was enhanced in T1D/AIT in IL1β- stimulated monocytes and after IL1β/1,25(OH)_2_D_3_ co-stimulation [IL1β_T1D/AIT vs HC_ p = 0.02; IL1β/1,25(OH)_2_D_3 T1D/AIT vs HC_ p = 0.001] compared to HC. Group-wise comparison between AD/AIT and HC revealed increased CCL-2 secretion after IL1β/1,25(OH)_2_D_3_ co-stimulation [IL1β/1,25(OH)_2_D_3 AD/AIT vs HC_ p = 0.02] and in IL1β-inflammatory stimulated monocytes by a trend (IL1β_AD/AIT vs HC_ p = 0.06).

### Vitamin D Effects on Anti-Inflammatory Gene Expression and Protein Secretion

#### PD-L1 and IL-10 mRNA Expression Enhanced Through Vitamin D Treatment

**Figure 4:**
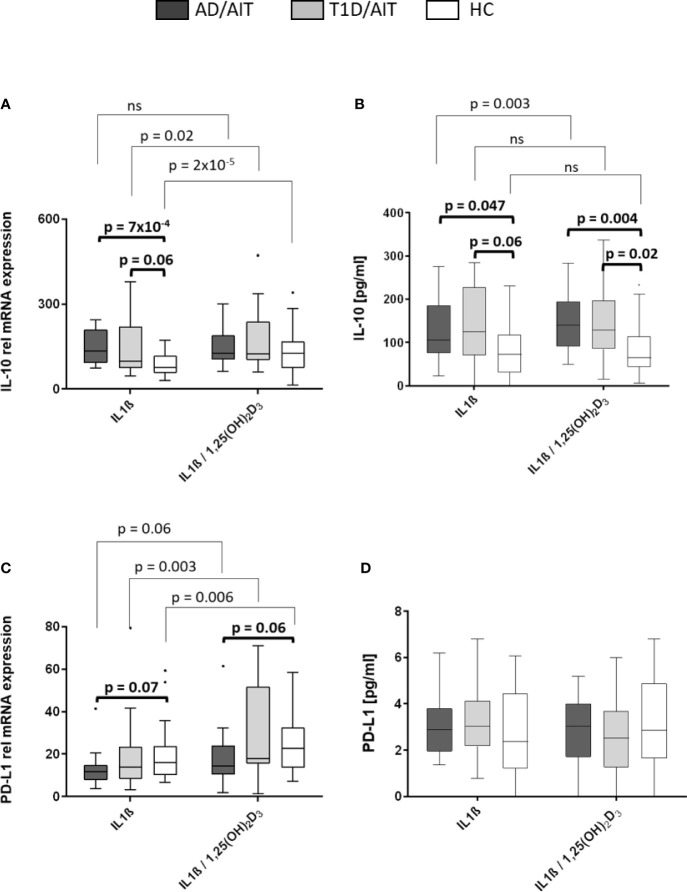
Vitamin D mediated enhancement of anti-inflammatory molecules IL-10 and PD-L1. Gene expression **(A, C)** and protein secretion **(B, D)** of anti-inflammatory cytokine IL-10 and immune regulator programmed death ligand 1 (PD-L1) after in-vitro 1,25(OH)_2_D_3_ administration in IL1β-activated monocytes of APS-2 patients and HC. Data are presented as median values obtained from 15 AD/AIT patients, 15 T1D/AIT patients and 30 healthy controls (HC). T1D, type 1 diabetes; AIT, autoimmune thyroiditis; AD, Addison’s disease; HC, healthy controls. Values ​​are considered to be statistically significant when p <0.05.

The mRNA expression levels of pro-inflammatory cytokines IL-10 and programmed death ligand 1 (PD-L1) was measured under basal conditions and after treatment with 1,25(OH)_2_D_3_. Vitamin D treatment of IL1β-activated primary human monocytes induced a significant increase of IL-10 mRNA expression in T1D/AIT patients and HC, whereas in AD/AIT patients no such increase in IL-10 expression was observed [T1D/AIT_IL1β vs IL1β/1,25(OH)2D3_ p = 0.02; HC_IL1β vs IL1β/1,25(OH)2D3_ p = 2x10^-5^] (**Figure 4A**). mRNA expression of PD-L1 also increased upon treatment with 1,25(OH)_2_D_3_ in IL1β-stimulated monocytes in HC as well as in both APS-2 patient cohorts [AD/AIT_IL1β vs IL1β/1,25(OH)2D3_ p = 0.006; T1D/AIT_IL1β vs IL1β/1,25(OH)2D3_ p = 0.003; HC_IL1β vs IL1β/1,25(OH)2D3_ p = 0.006]. Furthermore, unstimulated monocytes demonstrated increased PD-L1 expression in all cohorts upon 1,25(OH)_2_D_3_ addition (**Supplementary Material Table A1**). Group-wise comparison revealed striking differences of IL-10 expression between HC and APS-2 patients. In IL1β-activated monocytes, higher IL-10 levels were found in APS-2 patients than in HC (IL1β_AD/AIT vs HC_ p = 7x10^-4^, IL1β_T1D/AIT vs HC_ p = 0.06) which disappeared after vitamin D treatment. PD-L1 expression levels revealed a trend for lower PD-L1 levels in AD/AIT patients compared to HC in IL1β- and IL1β/1,25(OH)_2_D_3_ co-stimulated monocytes [IL1β_AD/AIT vs HC_ p = 0.07, IL1β/1,25(OH)_2_D_3 AD/AIT vs HC_ p = 0.06] (**Figure 4C**).

#### Increased IL-10 Secretion in APS-2 Patients

While IL-10 secretion was upregulated in IL1β-stimulated monocytes of AD/AIT patients after treatment with 1,25(OH)_2_D_3_ [IL-10_IL1β vs IL1β/1,25(OH)2D3_ p = 0.003], this enhancement was neither observed in T1D/AIT patients nor in HC (**Figure 4B**). Nevertheless, group differences were observed between APS-2 and HC. IL1β-stimulation in monocytes enhanced the IL-10 secretion in AD/AIT (IL1β_AD/AIT vs HC_ p = 0.047) also found as a trend in T1D/AIT compared to HC (IL1β_AD/AIT vs HC_ p = 0.06). The enhanced secretion of IL-10 was observed in both APS-2 cohorts compared to HC [IL1β/1,25(OH)_2_D_3 AD/AIT vs HC_ p = 0.004; IL1β/1,25(OH)_2_D_3 T1D/AIT vs HC_ p =_ _0.02]. No increase of IL1β-induced PD-L1 secretion was observed in either the APS-2 cohorts or in HC after addition of 1,25(OH)_2_D_3_ (**Figure 4D**). Only unstimulated monocytes demonstrated increased PD-L1 secretion upon 1,25(OH)_2_D_3_ addition in AD/AIT, T1D/AIT, and HC (**Supplementary Material Table A2**).

### Assessment of HLA αβ Heterodimers in APS-2 Cohorts and HC

The frequency of HLA DQ heterodimers was assessed for both APS-2 and HC cohorts and is shown in **Table 1**. DQA1/DQB1 Heterodimers 0501/0201 and 0501/0302 were more frequent in both APS-2 cohorts compared to controls: 0501/0201: AD/AIT OR 3.2; p = 0.02; T1D/AIT OR 4.0; p = 0.004; 0501/0302 AD/AIT and T1D/AIT OR 8.5; p = 0.03. However, the heterodimer 0501/0301 was less frequent in T1D/AIT (OR 0.05; p = 0.002) and in AD/AIT by a trend (OR 0.4; p = 0.1) compared to normal subjects. Both 0301/0201 and 0301/0302 DQA1/DQB1 heterodimers were more frequent in APS-2 patients. Here, 0301/0201 was more prevalent in AD/AIT (OR 3.8; p = 0.03), and in T1D/AIT (OR 3.2; p = 0.07); and 0301/0302 more frequent in T1D/AIT (OR 5.8; p = 0.002) and in AD/AIT group only showed a trend (OR 2.6; p = 0.1). Thus, the HLA DQ heterodimers differentiate between the two APS-2 cohorts AD/AIT and T1D/AIT.

**Table 1:** Frequencies of αβ HLA Heterodimers in APS-2 and HC. Only αβ Heterodimers with a frequency higher than 5% in our participants are displayed.

αβ HLA Hetero-dimers	Healthy controls (n = 30)	%	AD/AIT (n = 15)	%	Odds ratio (OR)	95% CI	p-value	T1D/AIT (n = 15)	%	Odds ratio (OR)	95% CI	p-value
0102/0201	6	5.0	2	3.3	0.78	0.15 4.18	0.7	0	0.0	0.28	0.04 2.04	0.21
0102/0602	4	3.3	4	6.7	2.07	0.51 8.39	0.3	0	0.0	0.21	0.03 1.78	0.15
0103/0603	10	8.3	1	1.7	**0.19**	**0.03 1.21**	**0.079**	3	5.0	0.57	0.16 2.16	0.41
0104/0501	6	5.0	0	0.0	**0.14**	**0.02 1.25**	**0.078**	1	1.7	0.32	0.04 2.48	0.27
0201/0201	7	5.8	0	0.0	**0.13**	**0.02 1.06**	**0.057**	0	0.0	**0.13**	**0.01 1.06**	**0.057**
0301/0201	4	3.3	7	11.7	**3.83**	**1.15 12.71**	**0.028**	6	10.0	**3.22**	**0.92 11.23**	**0.066**
0301/0301	11	9.2	3	5.0	0.52	0.14 1.91	0.32	2	3.3	0.34	0.08 1.50	0.15
0301/0302	4	3.3	5	8.3	2.63	0.71 9.80	0.14	10	16.7	**5.80**	**1.94 17.3**	**0.0017**
0301/0303	0	0.0	0	0.0	**-**	**-**	**-**	4	6.7	**19.19**	**2.5 146.2**	**0.0043**
0401/0402	2	1.7	4	6.7	**4.21**	**0.85 20.97**	**0.078**	1	1.7	1.00	1 1	1
0501/0201	7	5.8	10	16.7	**3.22**	**1.21 8.63**	**0.019**	12	20.0	**4.04**	**1.58 10.33**	**0.0036**
0501/0301	17	14.2	4	6.7	0.43	0.14 1.32	0.14	0	0.0	**0.05**	**0.007 0.33**	**0.0022**
0501/0302	1	0.8	4	6.7	**8.50**	**1.30 55.34**	**0.025**	4	6.7	**8.50**	**1.31 55.34**	**0.025**

Haplotypes with a higher frequency than 5% are displayed, DQA1 alleles are always mentioned before DQB1 alleles.

AD, Addison’s disease; T1D, type 1 diabetes; AIT, autoimmune thyroiditis.

Statistically significant values and trends are displayed in bold type.

#### Low-Risk HLA DQ Genotype Is Associated With Increased Expression of VDR, CYP27B1, CD14, and IL-10

**Figure 5:**
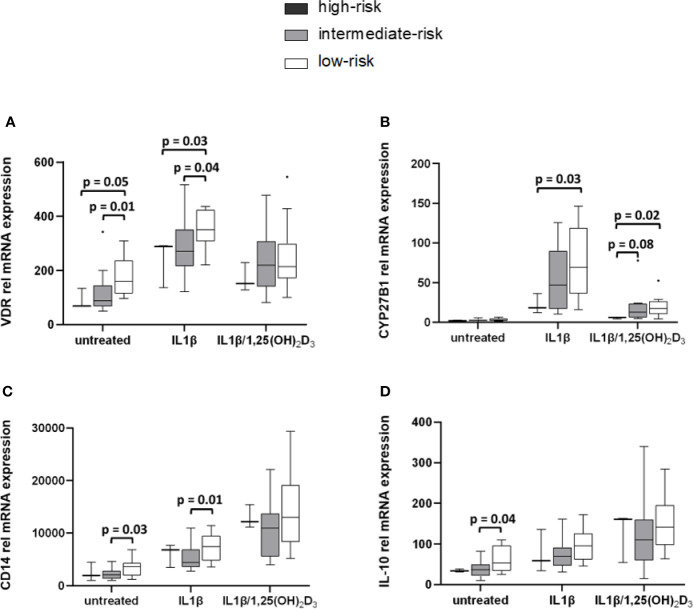
High-risk HLA affects VDR and CYP27B1 mRNA transcription levels. Gene expression levels of HC were stratified according to their HLA types. VDR **(A)** and CYP27B1 **(B)** gene expression of high-risk HLA and intermediate-risk HLA carriers are lowered compared to carriers with low-risk HLA. Low-risk HLA type is associated with increased CD14 **(C)** and IL-10 **(D)** expression in HC. HLA frequencies in HC: high-risk HLA (n = 3), intermediate-risk HLA (n = 13), low-risk HLA (n = 14).

Stratification of monocytic gene expression was performed for HLA DQ risk in healthy controls (**Figure 5**) and in APS-2 patients (**Supplementary Material Table A3**). VDR and CYP27B1 expression was reduced in healthy individuals with high-risk HLA (VDR_untreated_ p = 0.05, VDR_IL1β_ p = 0.03; CYP27B1_IL1β_ p = 0.03, CYP27B1_IL1β/1,25(OH)2D3_ p = 0.02) and intermediate-risk HLA (VDR_untreated_ p = 0.01, VDR_IL1β_ p = 0.04) compared to controls with low-risk HLA (**Figures 5A, B**). The HLA risk category was also associated with cytokine and CD14 gene modulation. CD14 expression was enhanced (CD14_untreated_ p = 0.03, CD14_IL1β_ p = 0.01) in healthy individuals with low-risk HLA as well as their IL-10 expression (IL-10_untreated_ p = 0.04) (**Figures 5C, D**).

## Discussion

Autoimmune disorders, including endocrinopathies are associated with genetic variants of the vitamin D system (26–31). The multiplicity of endocrinopathies in APS-2 suggests a more severe immune defect, involving a stronger genetic susceptibility. HLA DQ risk carriers may have a distinct vitamin D effect on monocytic cytokines since vitamin D response elements (VDRE) are located upstream from promoter regions of HLA class II genes (32).

CD14^+^ monocytes of HC and APS-2 patients demonstrated a 1,25(OH)_2_D_3_-induced negative feedback regulation of VDR and CYP27B1 and increased CYP24A1 expression, revealing a natural regulatory vitamin D circuit in both patients and HC. Vitamin D upregulated CD14 in both T1D/AIT patients and HC, in agreement with earlier reports (33, 34). Thereby vitamin D suppresses DC formation of monocytes (34, 35).

Patients’ monocytes express and secrete higher levels of inflammatory cytokines IL-6 and CCL-2. *In vitro* vitamin D treatment reduces IL-6, CCL-2, IL-23A, and IL-15 and enhances pro-inflammatory IL-10 and PD-L1 expression and secretion. AD/AIT patients displayed a lower expression of the pro-inflammatory cytokines and an increased production of anti-inflammatory IL-10, which can be attributed to their GC substitution. Both steroids vitamin D and GC act synergistically mediated by a co-activator complex (MED14) which enhances both VDR and glucocorticoid receptor action (36). This suggests, that a combined administration of vitamin D and GC could resolve inflammatory conditions and could potentially also reduce the need for higher GC doses to avoid GC side effects. For diseases like asthma and psoriasis this combined therapy has already been approved (37–40). The combination therapy with vitamin D and glucocorticoids therefore offers a new anti-inflammatory therapeutic approach for APS-2 patients.

The immune response to both vitamin D and to GC is variable and depends on genetic variants (27, 41, 42). We have identified specific epitopes of risk HLA alleles associated with APS-2 with the HLA-DQB1 position 57 defining disease susceptibility (25). We found HLA DQA1*0301:DQB1*0302 conferring a significantly increased risk for APS-type 1 diabetes and less for APS-Addison´s disease. Here we show that healthy carriers of αβ-HLA DQ heterodimers conferring protection, differ for IL-10, CD14 and vitamin D pathway gene expression. Protective HLA alleles (neither DQ2 nor DQ8) associate with higher levels of anti-inflammatory IL-10 and CD14, corresponding to a tolerogenic immune profile. In contrast high-risk HLA carriers showed a lower expression of VDR and CYP27B1 which implies less responsiveness to vitamin D. This corresponds to the finding that monocytes from subjects homozygous for HLA high-risk show a stronger surface expression of HLA-DR and -DQ, and also more IL-1β and IFN-γ (22). Furthermore newborns with high-risk HLA DR4-DQ8 have less potential for Th2 differentiation with reduced CCR4-, IL-13 and Th2-inducing transcription factor GATA-3 levels (43). This could suggest that tailored vitamin D supplementation based on the HLA genotype aimed to modulate monocytic cytokine responses in such individuals. Large scale genetic and epigenetic fine mapping in 21 autoimmune diseases identified causal variants primarily in regulatory elements that—amongst others—enhance antigen processing for MHC presentation (44). Thereby autoimmune susceptibility is two-tiered. An altered cytokine profile can enhance the expression of HLA molecules leading to enhanced autoantigen presentation in carriers of high-risk HLA. The combined genetic and epigenetic risk can be targeted by vitamin D that suppresses HLA-DR and CD4 antigen expression with autocrine downregulation of T cells (21). This effect can be relevant for primary prophylaxis as shown in a controlled trial of Influenza vaccination of elderly individuals where adjuvant vitamin D shifts the TH1-cytokine response towards a more immunosuppressive state with increased levels of TH2 cytokines IL-4, IL-5, IL-10 (45).

Due to the limited numbers in our investigation these findings need to be confirmed in larger cohorts. The molecular mechanisms for HLA DQ allele specific vitamin D effects need to be addressed by upstream sequencing and functional studies for an individualized vitamin D therapy. Nevertheless, our study shows that individuals at high genetic risk develop an amplified monocytic cytokine response that can be modulated by vitamin D.

## Data Availability Statement

The datasets presented in this study can be found in online repositories. The names of the repository/repositories and accession number(s) can be found in the article/**Supplementary Material**. 

## Ethics Statement

The studies involving human participants were reviewed and approved by the Ethics Committee of the University Hospital Frankfurt am Main (Registration Number: 247/05). The patients/participants provided their written informed consent to participate in this study. 

## Author Contributions

AK designed, performed and evaluated experiments, wrote the manuscript, and recruited participants. MP-M and KB conceived and supervised the work, corrected the manuscript, and contributed equally to this work. FS performed and supervised experiments. GM recruited participants. All authors contributed to the article and approved the submitted version. 

## Funding

This work was supported by the Else Kröner-Fresenius Foundation (EKFS), Research Training Group Translational Research Innovation-Pharma (TRIP) and project funds from the German Diabetes Society (Deutsche Diabetes Gesellschaft-DDG).

## Conflict of Interest

The authors declare that the research was conducted in the absence of any commercial or financial relationships that could be construed as a potential conflict of interest.

## Abbreviations

AD, Addison’s disease; T1D, type 1 diabetes; AIT, autoimmune thyroiditis; APS-2, autoimmune polyglandular syndrome type 2; HC, healthy controls; CYP27B1, 1-α-hydroxylase; VDR, vitamin D receptor; GC, glucocorticoid; 25(OH)D_3_, 25-hydroxyvitamin D_3_; 1,25(OH)_2_D_3_, 1,25-dihydroxyvitamin D_3_; DC, dendritic cell.
