# Supplementary material for: Monocytic Cytokines in Autoimmune Polyglandular Syndrome Type 2 Are Modulated by Vitamin D and HLA-DQ
Source: Front Immunol. 2020 Dec 7;11:583709. doi: 10.3389/fimmu.2020.583709 (PMC7750404; doi:10.3389/fimmu.2020.583709)
Supplement: Supplementary file 1 [file Table_1.docx]

**Supplementary Material**

**Table A1:** Effect of 1,25(OH)_2_D_3_ treatment on mRNA expression levels of vitamin D pathway genes, monocyte marker CD14, pro-, and anti-inflammatory cytokines in CD14^+^ monocytes of Healthy controls, AD/AIT patients and T1D/AIT patients.

**Table A2:** Effect of 1,25(OH)_2_D_3_ treatment on pro-, and anti-inflammatory cytokine secretion in CD14^+^ monocytes of Healthy controls, AD/AIT patients and T1D/AIT patients

**Table A3:** Association of HLA DQ risk category on gene expression levels in HC, AD/AIT and T1D/AIT patients
